# Comparison of individual and pooled urine samples for estimating the presence and intensity of *Schistosoma haematobium* infections at the population level

**DOI:** 10.1186/s13071-015-1205-7

**Published:** 2015-11-16

**Authors:** Abraham Degarege, Berhanu Erko, Zeleke Mekonnen, Mengistu Legesse, Yohannes Negash, Jozef Vercruysse, Bruno Levecke

**Affiliations:** Aklilu Lemma Institute of Pathobiology, Addis Ababa University, Addis Ababa, Ethiopia; Department of Epidemiology, Robert Stempel College of Public Health and Social Work, Florida International University, Miami, FL USA; Department of Medical Laboratory Sciences and Pathology, College of Public Health and Medical Sciences, Jimma University, Jimma, Ethiopia; Department of Virology, Parasitology and Immunology, Faculty of Veterinary Medicine, Ghent University, Merelbeke, Belgium

**Keywords:** *Schistosoma haematobium*, Pooling, Infection intensity, Sensitivity, Mass drug administration, Monitoring and evaluation, Ethiopia

## Abstract

**Background:**

There is a lack of cost-effective diagnostic strategies to evaluate whether mass drug administration (MDA) programmes to control *Schistosoma haematobium* progress as anticipated. The purpose of this study is to provide a proof-of-principle for examination of pooled urine samples as a strategy for rapid assessment of presence and intensity of *Schistosoma haematobium* infections at the population level.

**Methods:**

A total of 640 urine samples were collected from 520 school-aged children (520 at baseline and 120 at follow-up) during a clinical trial that was designed to assess the efficacy of praziquantel against *Schistosoma haematobium* infections in Ethiopia. Individual and pooled urine samples were screened using the filtration technique (volume of 10 ml urine) to determine the number of *S. haematobium* eggs in 10 ml of urine. Samples were pooled into pools of 5 (*n* = 128), 10 (*n* = 64) and 20 (*n* = 32) individual samples. The sensitivity, the probability of finding at least one egg in a pooled sample when the mean urine egg count (UEC) of the corresponding individual urine samples was not zero, was calculated for each pool size. UECs of a pooled examination strategy were compared with the mean UECs of the corresponding individual samples.

**Results:**

The sensitivity of a pooled examination strategy was 50.6 % for pools of 5, 68.6 % for pools of 10 and 63.3 % for pools of 20. The sensitivity of a pooled examination strategy increased as a function of increasing mean UEC of the corresponding individual urine samples. For each of the three pool sizes, there was a significant positive correlation between mean UECs of individual and those obtained in pooled samples (correlation coefficient: 0.81 – 0.93). Examination of pools of 5 provided significantly lower UECs compared to the individual examination strategy (3.9 eggs/10 ml urine versus 5.0 eggs/10 ml urine). For pools of 10 (4.4 eggs/10 ml) and 20 (4.2 eggs/10 ml), no significant difference in UECs was observed.

**Conclusions:**

Examination of pooled urine samples applying urine filtration holds promise for rapid assessment of intensity of *S. haematobium* infections, but may fail to detect presence of infections when endemicity is low. Further investigation is required to determine when and how pooling can be optimally implemented in monitoring of mass drug administration programmes.

## Background

Neglected tropical diseases (NTDs), which are caused by a variety of viruses, bacteria and parasites, pose an important burden on public health in several parts of the world. NTDs are the fourth most important group contributing to the global burden attributable to communicable diseases, accounting for ~48 million disability-adjusted life years lost each year, mainly affecting people in developing countries [1, 2].

Recently, the World Health Organization (WHO) has set the ambitious targets to control, eliminate and eradicate 10 specific NTDs by 2020, including urinary schistosomiasis [3]. These targets were subsequently endorsed in the London Declaration on NTDs (January, 2012), and supported by more than 70 pharmaceutical companies, governments and global health organizations by sustaining or expanding NTD drug donation programmes. For example, the number of tablets donated for the control of schistosmiassis from Merck KGaA company increased from ~20 million tablets in 2012 to 250 million annually for an unlimited period [4]. Since the London Declaration, more than 5.5 billion tablets have been donated [5].

Although this is probably the largest public health drug donation programme in the world, it also creates the need to adequately monitor these mass drug administration (MDA) programmes to verify whether the targets set are being met, and if necessary, to adjust the programme implemented. Currently, the impact of MDA programmes is evaluated by re-assessing the epidemiology (prevalence and intensity of infections for some NTDs) through large-scale epidemiological surveys in which subjects are individually screened. However, this strategy of individually screening a large number of subjects faces some important financial and technical obstacles, particularly when MDA programmes are mainly operating in resource-limited settings. In veterinary medicine, pooling samples has been applied to reduce number of diagnosis, and hence reducing the costs for monitoring the impact of measures to control helminth infections [6–8]. Recently, pooling stool samples has also been evaluated for the assessment of two NTDs, including soil-transmitted helminthiasis and schistosomiasis (*Schistosoma mansoni*) [9, 10]. The results indicated that examination of pooled stool samples did not compromise on the accuracy of the assessment of the intensity of soil-transmitted helminth and *Schistosoma mansoni* infections, while reducing the time in the laboratory by at least 70 % [10]. These findings suggest that pooling samples could result in important cost-savings in large-scale epidemiological surveys. This is particularly when these NTDs are often heterogeneously scattered across large geographical areas [11]. Today, it remains unclear whether pooling would also be a reliable strategy to assess other NTDs that are diagnosed in other specimens. The purpose of this study is to provide a proof-of-principle for examination of pooled urine samples as a strategy for rapid assessment of the presence and the intensity of *S. haematobium* infections at the population level.

## Methods

### Study area and population

The study was conducted in Middle Awash Valley of the Afar Region (northeastern Ethiopia) between February and May 2014, including four Afar ethnic villages (Anbesh, Buri, Hassabo and Hanledebe) of the Amibara District. These villages are located 300 to 350 km from the capital Addis Ababa at an altitude of approximately 725 m above sea level. The annual rainfall is on average 654 mm^3^, the average temperature is 25.6 °C. Previous studies in Buri and Hassabo villages reported a prevalence of urinary schistosomiasis ranging from 24.5 to 47.6 % in children [12, 13]. The current study focused on school-aged children, all children between 5 and 16 years of age being eligible for the study.

### Study design

This study was part of a multi-country trial designed to assess the efficacy of a single dose of praziquantel (40 mg/kg) against *Schistosoma* spp. Infections in school-aged children, including *Schistosoma mansoni* (Cameroon, Ethiopia, Mali, and Tanzania), *S. haematobium* (Cameroon, Ethiopia, Mali, and Tanzania) and *S. japonicum* (the Philipines). For this trial, the initial aim was to enroll, at least 125 infected children per *Schistosoma* species at each study site. As a secondary objective, the proof-of-principle for examination of pooled urine samples as a strategy for rapid assessment of presence and intensity of *S. haematobium* infections was evaluated in Ethiopia. To this end, eggs of *S. haematobium* eggs were quantified in urine samples collected during the pre-intervention and post-intervention applying both an individual and a pooled examination strategy. In the past, pools of 10, 20 and 60 have been applied for quantifying eggs in soil-transmitted helminthiasis in stool. However, due to a more focal distribution of schistosomiasis, which may result in more false negative test results when too many samples are pooled, we opted for pooling into pools of 5, 10 and 20 [10].

### Field and laboratory procedures

The headmasters of the schools and village administrators were informed about the aim of the study. Children, who were not attending school, were brought to the school by the village administrator. Children who agreed to participate in the study were asked to bring their parents to the school. The field team, the head master and the village administrators then explained the purpose of the study in more detail to both the children and the parents. Only those children who were willing to participate and whose parents approved their participation in the study were given labeled plastic containers (200 ml capacity) in order to provide urine samples. Urine samples were collected between 10:00 am and 2:00 pm. For each child, two vials containing 0.1 formalin (37 % formaldehyde) were subsequently filled with 10 ml urine. The first vial was individually processed applying the urine filtration technique one week after sample collection [14]. For the filtration of urine we used polycarbonate membrane filters of 13 mm diameter and 12 to 14 μm pore size (Sterlitech, Kent, WA, United States of America)), the second vial was used to make pools of urine.

**Fig. 1:**
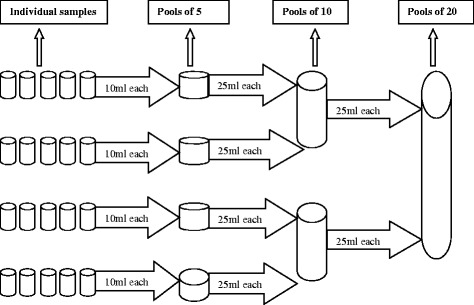
The procedure to obtain pools of 5, 10 and 20 individual samples

Urine samples were pooled by adapting a cascade system that had been previously described for stool samples [9, a visual tutorial is available at https://www.youtube.com/watch?v=IUZijtBABn0)]. In the past, pools of 10, 20 and 60 have been applied for quantifying eggs of soil-transmitted helminths in stool samples [9]. However, due to a more focal distribution of schistosomiasis, which may result in more false negative test results when too many samples are pooled, we opted to pool individual urine samples into pools of 5, 10 and 20. Figure 1 describes the procedure to pool urine samples. Pools of 5, 10 and 20 individual samples were made applying the following procedures. First, plastic vials containing urine samples in rows of 5 samples were arranged according to the unique subject identifier (increasing order). Assuming that there is no correlation between this subject identifier and the urine egg counts (UECs), we assumed that the samples were randomly arranged. Then, 10 ml from each vial of the same row was transferred to a 50 ml vial to prepare pools of 5 individual samples. Afterwards, 25 ml urine from each of two different vials containing pooled samples of 5 individuals was transferred (after shaking) to another 50 ml vial to produce pools of 10 individual samples. Finally, 25 ml urine from each of two different vials containing pooled samples of 10 individuals was transferred (after shaking) to another 50 ml size vial to produce pools of 20 individual samples. Finally, 10 ml of each of the pooled urine samples was filtered and examined using the same procedure as used for individual samples.

Fourteen to 21 days post-intervention, urine samples from subjects excreting eggs at the pre-intervention survey were again collected and processed applying the same procedures during the pre-intervention survey. Eggs of *S. haematobium* were quantified in 10 ml of both individual and pooled urine samples within 1 week after collecting the samples applying the urine filtration technique.

### Statistical data analysis

We compared both the qualitative (sensitivity) and the quantitative (UECs) diagnostic performance of a pooled examination strategy at the pooled level. Sensitivity, the probability of finding at least one egg in a pooled sample when the mean UECs of the corresponding individual urine samples was not zero, was calculated for each pool size. To this end, we assumed a specificity of 100 % for both strategies. We explored the variation in sensitivity for each pool size by a logistic regression model. To do this, we set the pooled test result (positive/negative) as the outcome, and the mean UECs of the corresponding individual urine samples as covariate. The predictive power of these models was evaluated by the proportion of the observed outcome that was correctly predicted by the model. For this, an individual probability >0.5 was set as a positive test result, and negative if different. Finally, the sensitivity for each of the observed values of mean UECs was calculated based on these models.

The agreement in UECs across the three methods was verified by a permutation test (10,000 iterations) based on Pearson correlation coefficient and differences in UECs. The Tukey’s method was applied for pairwise comparison. The level of significance was set at *p* < 0.05.

### Ethical consideration

The study was approved by the Institutional Review Board of Aklilu Lemma Institute of Pathobiology, Addis Ababa University (Ref. No. IRB/22-A/2012/13). The District Health Office, school authorities, teachers, parents, and the children were informed about the purpose and procedures of the study. Only those children who were willing to participate and from whom the parents or guardian gave verbal informed consent were included in the study. As the study population was mainly illiterate the Institutional Review Board of Aklilu Lemma Institute of Pathobiology indorsed oral consenting of the parents or guardians of the children. Since the study involved minimal risk the Institutional Review Board did not require tape recording or any other form of proof of the informed consent processes. Children who were infected with *S. haematobium* infection were treated with praziquantel (40 mg/kg body weight).

## Results

### Prevalence and infection intensity

**Fig. 2:**
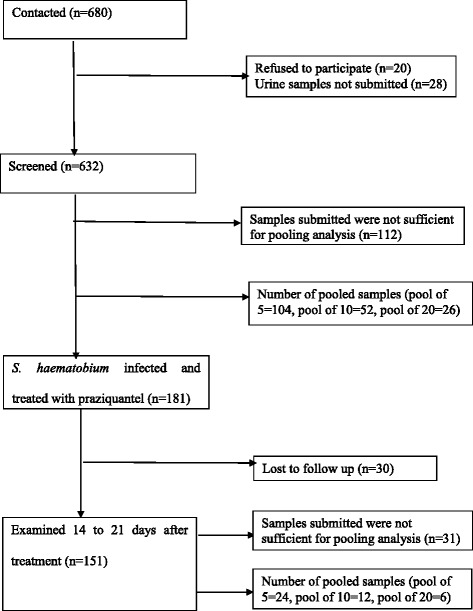
Number of the study subjects enrolled, and number of pooled samples included in the analysis

A total of 632 children (mean age = 9.8 years, female/male ratio = 0.72) at the pre-intervention survey and 151 children during the post-intervention were screened individually for *S. haematobium* infections (Fig. 2). A total of 640 samples, including 520 samples at pre-intervention and 120 samples at post-intervention, were also analysed using the pooling strategy. When samples were individually examined, *S. haematobium* eggs were found in 181 out of 640 urine samples (28.3 %). The number of positive pools was 84/128 (65.6 %), 51/64 (79.7 %) and 30/32 (93.6 %) for pools of 5, 10 and 20 individual stool samples, respectively. The arithmetic mean of UECs equaled 5.0 eggs/10 ml, with individual UECs ranging from 0 to 426 eggs/10 ml. All the 120 samples at post-intervention were negative for *S. haematobium* eggs. The arithmetic mean UEC of the 640 samples based on examination of pools of 5, pools of 10 and pools of 20 was 3.9 eggs/10 ml urine, 4.4 eggs/10 ml and 4.2 eggs/10 ml, respectively.

### Sensitivity

**Fig. 3:**
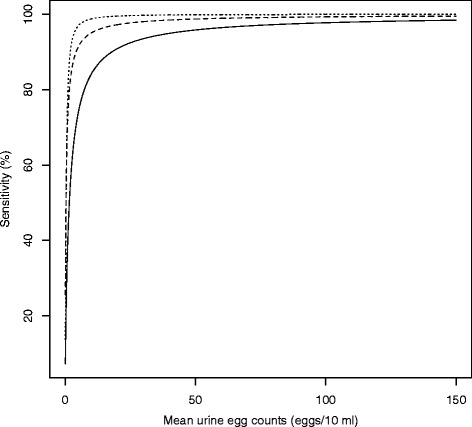
The predicted sensitivity derived from logistic regression for different pool sizes. Pools of 5 samples are represented by the straight line, pools of 10 samples by the dashed line, and pools of 20 samples by the dotted line

Eggs were not found in pools for which the mean of individual UECs equaled zero (i.e. sensitivity of the individual examination strategy equaled 100 %). The sensitivity of a pooled examination strategy is summarized in Table 1. The pooled examination strategy was significantly less sensitive (pools of 5 = 53.9 %, 95 % confidence intervals (CI) [40.0, 61.2]; pools of 10 = 68.7 %, 95 % CI [55.9, 81.4]; pools of 20 = 63.3 %, 95 % CI [46.1, 80.6]). However, the sensitivity of the pooling examination strategy increased significantly as a function of the mean of UECs of the corresponding individual samples. The pooled examination strategy was equally sensitive with the individual examination strategy when the UECs became high (Fig. 3). The models correctly predicted the observed test results in 72.9 % for pools of 5, 78.4 % pools of 10 and 83.3 % for pools of 20.

**Table 1:** The number of positive *Schistosoma haematobium* samples and the sensitivity of the different pool sizes

Pool size	Sample size	Number of positive samples (%)	Sensitivity (%) (95 % CI)
1	640	181 (28.3 %)	100 %
5	128	84 (65.6)	50.6 (40.0; 61.2)
10	64	51 (79.7)	68.6 (55.9; 81.4)
20	32	30 (93.6)	63.3 (46.1; 80.1)

### Infection intensity

**Fig. 4:**
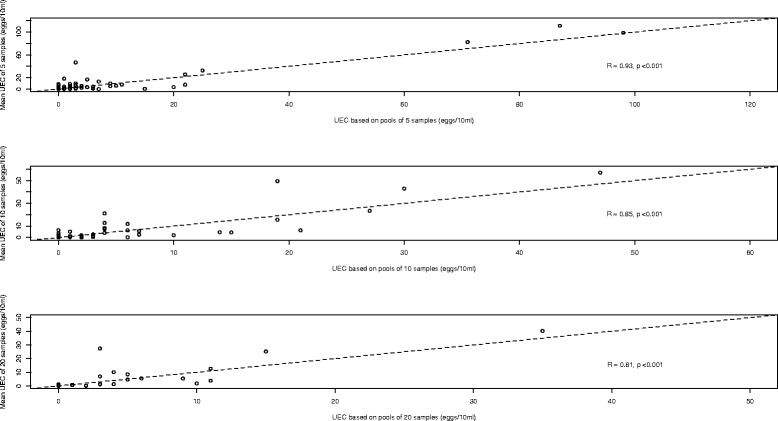
The agreement in urine egg counts between an individual and pooled examination strategy. Each of the 3 scatter plots represents the agreement in mean egg counts per 10 ml of individual urine samples and egg counts of pooled urine samples. The plots represent pool sizes of 5, 10 and 20. The magnitude of correlation for each plot is based on the Pearson correlation coefficient

Overall, there was a positive significant correlation between the UECs obtained by examining pooled samples and the mean UECs of the corresponding individual urine samples (Fig. 4). The correlation coefficient (R) was the lowest for pools of 20 (R = 0.81, *p* <0.001), and the highest for pools of 5 (R = 0.93, *p* <0.001). Examination of pools of 5 provided significantly lower UECs compared to an individual examination strategy (mean difference = −1.08 eggs/10 ml, 95 % CI [−2.2, −0.15]). For pools of 10 (mean difference = −0.61 eggs/10 ml; 95 % CI [−2.2, 0.79] and 20 (mean difference = −0.85 eggs/10 ml; 95 % CI [−2.92, 0.77]), no significant difference in UECs was observed (Table 2).

**Table 2:** Pair-wise comparison of arithmetic mean urine egg count between individual and a pooled examination strategy

Pool size	Sample size	Mean UEC (eggs/10 ml) (95 % CI)	Level of significance for pair-wise comparison
1	640	5.0 (3.2; 7.2)	_
5	128	3.9 (1.9; 6.5)	0.032
10	64	4.4 (2.5; 6.7)	0.44
20	32	4.2 (2.2; 6.8)	0.44

UEC: urine egg count

## Discussion

To date there are no cost-effective tools to verify whether MDA programmes progress as anticipated, and if necessary, to adjust the programme implemented. The present study provides a proof-of-principle for examination of pooled urine samples as a strategy for rapid assessment of *S. haematobium* infections, by evaluating the diagnostic performance of pooling urine for both the qualitative (presence of infection) and quantitative assessment (infection intensity) of *S. haematobium* infections at the population level. In the current study, the sensitivity of a pooled examination strategy was low (50.6 % - 68.6 %), but increased as a function of increasing mean UEC of the corresponding individual urine samples. For each of the three pool sizes, there was a significant positive correlation between mean UECs of individual and those obtained in pooled samples (correlation coefficient: 0.81 – 0.93). A significant difference in UEC was observed only for pools of 5, resulting in significantly lower UECs compared to the individual examination strategy (3.9 eggs/10 ml urine versus 5.0 eggs/10 ml urine).

These results indicate that pooling urine samples lacks sensitivity to detect low levels of infection intensity (1–49 egg/10 ml urine, Fig. 4), and hence applying this strategy may result in falsely declaring a population free of disease where endemicity is low. This observation is not unexpected, and can be partially explained by a combination of a dilution effect when pooling samples and the intrinsic lack of sensitivity of urine filtration to detect low egg counts [15, 16]. To further minimize the probability of falsely declaring a population free of disease one may either consider examining more pooled urine (i.e. filtering 30 ml of pooled urine instead of 10 ml) or applying a more sensitive method, such as *S. haematobium* DNA or antigen detection methods [15, 16]. For example, in this setting it is expected that examining 30 ml of a pool of 20 individual samples instead of 10 ml would already increase the sensitivity from 63.3 to 95.1 % (= 1-(1–0.633)^3^). The quantitative performance highlights that pooling provides comparable estimates of infection intensity, and this finding is in line with previous studies assessing pooling of stool samples as an alternative strategy to evaluate helminth infections in both animals [6–8] and humans [9, 10], highlighting that pooling of urine samples could translate into important cost-savings in large-scaled epidemiological surveys required to monitor the progress of MDA programmes.

However, there are a few aspects that require further attention for *S. haematobium*. First, we only applied one diagnostic method and the sample size from the different villages was not sufficient to make powerful analysis for each area separately. Complementary field and/or in silicon studies evaluating pooling of urine samples in varying scenarios of endemicity of *S. haematobium*, and both diagnostic (the number of samples pooled, the volume of urine examined and the sensitivity of the diagnostic technique) and sampling efforts (number of urine samples examined per subject) are required to decide when and how to apply pooling of urine samples. For example, a mathematical framework for soil-transmitted helminths has recently been developed allowing health-care decision makers to adapt their survey design according to local epidemiology (level of aggregation and intensity of worm infections) and both diagnostic and sampling effort [17, an online tool for this framework can be found at https://paradesign.shinyapps.io/paradesign/].

Second, pools were made through a cascade system (pools of 20 were made out of pools of 10, pools of 10 were made out of pools of 5, and pools of 5 were made out of individual samples). Although this allowed evaluating different pools sizes with a minimum of amount of urine per individual (10 ml), it is not recommended when applied in a MDA programme. It would have major logistical issues, particularly when the number of samples to be pooled is ≥10 and the number of pools is large. In addition, it may introduce bias in UEC results as pools of 10 or 20 are not entirely independent of the pools of 5, and as the contribution of individual urine samples to the pools reduces as a function of pool size (the total volume of the pools was 50 ml across the pool sizes, and hence the contribution of the individual samples is 10 ml for pools of 5, 5 ml for pools of 10 and 2.5 ml for pools of 20). This difference in contribution of individual samples to the pool may explain the current result, where the correlation between UECs obtained by examining pooled samples and the mean UEC of corresponding individual samples was lowest for pools of 20 and highest for pools of 5. To avoid this potential bias in UECs it would be recommended to pool fixed volumes of individual samples for each pool size as we did for pools of 5 rather than using pools of 5 to make pools of 10 and pools of 10 to make pools of 20.

Third, from Table 1 it is clear that the proportion of pools containing eggs increases as a function of the number of samples pooled, and hence do not provide an accurate estimate of true underlying prevalence. Various researchers have developed statistical methods to estimate the true underlying prevalence based on the examination of pooled samples, but these need to be further validated for *S. haematobium* [17–19].

Finally, a cost-benefit analysis is highly recommended to verify whether pooling of urine samples in a large-scale epidemiological survey of *S. haematobium* infection is indeed cost-saving. This will help to guide programme managers and healthcare decision-makers in designing the most cost-effective survey to monitor MDA programmes aimed in the control of *S. haematobium* infection. To this end, cost estimates of preparing and analysing pooled urine samples should be assessed [10].

## Conclusions

To conclude, examination of pooled urine samples applying urine filtration holds promise for rapid assessment of intensity of *S. haematobium* infections, but may fail to detect presence of infections when endemicity is low. Further research is required to recommend when (different levels of endemicity; different phases of programme: control *vs.* elimination) and how (number of samples collected per subject, number of samples examined per pool, the number of individual samples pooled, the volume of urine examined and the sensitivity of the diagnostic technique) pooling urine samples could translate into important cost-savings in large-scaled epidemiological surveys to monitor progress of MDA programmes.
